# Implementation of PM-JAY in India: a qualitative study exploring the role of competency, organizational and leadership drivers shaping early roll-out of publicly funded health insurance in three Indian states

**DOI:** 10.1186/s12961-023-01012-7

**Published:** 2023-06-27

**Authors:** Swati Srivastava, Maria Paola Bertone, Sharmishtha Basu, Manuela De Allegri, Stephan Brenner

**Affiliations:** 1 Heidelberg Institute of Global Health, Medical Faculty and University Hospital, Heidelberg University, Im Neuenheimer Feld 130.3, 69120 Heidelberg, Germany; 2 Institute for Global Health and Development, Queen Margaret University, Edinburgh, EH21 6UU United Kingdom; 3 Deutsche Gesellschaft für Internationale Zusammenarbeit (GIZ) GmbH, B – 5/1 & 5/2 Ground Floor, Safdurjung Enclave, 110029 New Delhi, Delhi India

**Keywords:** National health insurance, Implementation, Process, Context, India

## Abstract

**Background:**

The Pradhan Mantri Jan Arogya Yojana (PM-JAY), a publicly funded health insurance scheme, was launched in India in September 2018 to provide financial access to health services for poor Indians. PM-JAY design enables state-level program adaptations to facilitate implementation in a decentralized health implementation space. This study examines the competency, organizational, and leadership approaches affecting PM-JAY implementation in three contextually different Indian states.

**Methods:**

We used a framework on implementation drivers (competency, organizational, and leadership) to understand factors facilitating or hampering implementation experiences in three PM-JAY models: third-party administrator in Uttar Pradesh, insurance in Chhattisgarh, and hybrid in Tamil Nadu. We adopted a qualitative exploratory approach and conducted 92 interviews with national, state, district, and hospital stakeholders involved in program design and implementation in Delhi, three state capitals, and two anonymized districts in each state, between February and April 2019. We used a deductive approach to content analysis and interpreted coded material to identify linkages between organizational features, drivers, and contextual elements affecting implementation.

**Results and conclusion:**

PM-JAY guideline flexibilities enabled implementation in very different states through state-adapted implementation models. These models utilized contextually relevant adaptations for staff and facility competencies and organizational and facilitative administration, which had considerable scope for improvement in terms of recruitment, competency development, programmatic implementation support, and rationalizing the joint needs of the program and implementers. Adaptations also created structural barriers in staff interactions and challenged implicit power asymmetries and organizational culture, indicating a need for aligning staff hierarchies and incentive structures. At the same time, specific adaptations such as decentralizing staff selection and task shifting (all models); sharing of claims processing between the insurer and state agency (insurance and hybrid model); and using stringent empanelment, accreditation, monitoring, and benchmarking criteria for performance assessment, and reserving secondary care benefit packages for public hospitals (both in the hybrid model) contributed to successful implementation. Contextual elements such as institutional memory of previous schemes and underlying state capacities influenced all aspects of implementation, including leadership styles and autonomy. These variations make comparisons across models difficult, yet highlight constraints and opportunities for cross-learning and optimizing implementation to achieve universal health coverage in decentralized contexts.

## Background

In low- and middle-income countries, publicly funded health insurance (PFHI) schemes have been promoted to advance universal health coverage (UHC) by improving financial access and financial health protection [1]. PFHI schemes are rather complex to implement, as they require adjustments across various functions related to beneficiary enrollment, contracting of private and public providers, financial claim management, and provider reimbursement [2].

Emerging evidence suggests that design choices and implementation strategies influence the success of a PFHI scheme within a given context [2]. Relevant contextual features to be addressed during PFHI implementation include aspects of governance (e.g., political will, organizational culture, and regulatory environment), available management capacities (e.g., financial and human resources defining monitoring, supervision, coordination, and communication), and societal buy-in (e.g., level of solidarity across social groups) [2–5]. Learning from different implementation experiences can therefore increase our understanding of how contextual features can facilitate or hinder successful PFHI roll-out.

In September 2018, the Government of India launched the largest-ever PFHI, the Pradhan Mantri Jan Arogya Yojana (PM-JAY) by subsuming existing state-level PFHI under a single national umbrella [6]. Under the scheme, beneficiaries present to hospitals, where their eligibility is verified through government databases, and they can be admitted for approximately 1400 inpatient procedures, for 3 days for pre-hospitalization services, and for 15 days for post-hospitalization services. Hospitals providing services under PM-JAY are empanelled under the scheme after meeting prescribed infrastructure and human resource criteria. PM-JAY differs from earlier Indian PFHI by expanding financial coverage to INR 500,000 per household annually, an amount that exceeded coverage of all previous schemes and expanded eligibility to nearly 500 million Indians, which is the largest population coverage thus far by any Indian PFHI. PM-JAY also enables utilization of services by beneficiaries across hospitals in all states and not only their states of domicile, which was lacking in previous PFHI. Further, PM-JAY is administered by the National Health Authority (NHA) at the federal level; state-level implementation, including hospital empanelment and reimbursement of claims, is overseen by state health agencies (SHAs), alone or in collaboration with different support agencies, depending on each state’s specific implementation strategy. This is aligned with state governments having legislative authority in health matters, including implementation of health programs, as per the Indian constitution. State-specific implementation strategies differ on the basis of state-level health system and administrative structures, prior PFHI implementation experiences, inpatient care and insurance capacities, and characteristics and distribution of the beneficiary population.

Implementation science provides different approaches to analyze health reforms, including PFHI, which are useful for understanding the program model, theory of change, target population characteristics, and potential alternative approaches. There is little synthetic evidence on the application of implementation science frameworks [7, 8], especially applied to PFHI. Early studies on PM-JAY implementation indicate a need to increase the responsiveness of the scheme for beneficiaries [9]; expand the technical, managerial, and leadership competencies of all staff involved in implementation [10], especially in private hospitals [9]; and examine the functioning of implementation models in different states and contexts [11]. In China, the integration of separate schemes for rural and urban residents in 2009 resulted in unified enrollment, regulation, and management structures, thereby streamlining scheme organization, administration, efficiency, and potentially, health outcomes and equity [12]; other studies have highlighted low competencies of doctors, governance issues, and misaligned incentive mechanisms to adversely affect performance [13, 14]. In Nigeria, low awareness of UHC policies, inadequate implementation capacities, and poor accountability mechanisms among all stakeholders were barriers for the implementation of the National Health Act [15–17]. In more resourceful settings such as the USA, the implementation of the Affordable Care Act of 2010 (Obamacare) was extensively delegated to state governments, resulting in numerous state adaptations and potential for political and leadership opposition and variable policy outcomes [18].

The approach taken for the roll-out of PM-JAY, i.e., using state-adapted strategies for implementation of a nationwide scheme, offers the opportunity to examine and compare different implementation strategies, as well as to explore whether and how programmatic challenges and adaptations might be linked to a given implementation context. This study examines the competency, organizational, and leadership approaches driving early PM-JAY implementation in contextually different settings.

## Methods

### Study setting: State selection and related PM-JAY implementation models

The PM-JAY implementation model is outlined in guidelines containing details of core features and potential state-level implementation adaptations that individual states can espouse. In this study, we focus on three states (Uttar Pradesh, Chhattisgarh, and Tamil Nadu), which each followed a different implementation approach.

Uttar Pradesh lies in northern India and is India’s most populous state, with approximately 241 million persons in 75 districts [19]. It had a Human Development Index (HDI) of 0.592 (medium) in 2021 [20]. Due to its large, growing, and primarily rural agricultural population, it has been a focus state of the Indian national government for social programs [21]. Chhattisgarh lies in central India and had an HDI of 0.605 (medium) in 2021 [20]. The state is rich in mineral and natural resources and has a large Adivasi (tribal) and primarily rural population of 29 million [19]. Tamil Nadu lies in southern India and has a population of 75 million [19]. It is one of India’s most progressive states, with an HDI of 0.686 in 2021 [20]. It is also one of the most urbanized states, with the second-largest economy of all Indian states [22]. Traditionally, political leadership in Uttar Pradesh has not given high priority to health programs [21], while Tamil Nadu has a long history of investments in social and health programs [23]. Since the late 1960s, Chhattisgarh has suffered from a long, violent conflict with left-wing Maoist groups organizing terrorist attacks against the government for independence and control of natural resources in tribal and remote districts, although this has subsided in recent times [24].

We first describe PM-JAY core design followed by state-specific implementation models.

#### PM-JAY core design

PM-JAY national guidelines and the individual contractual “Memorandum of Understanding” signed between the federal government and each state outline the roles and responsibilities of the NHA, SHAs, and district administration. Together, these define the key administrative and technical functions required for information and data management, content and prices of provided care, identification and verification of beneficiaries, and contracting of hospitals, insurers, and other agencies. States are expected to regulate and implement these functions, while institutional entities and structures for supervision and execution can vary. SHAs as public non-profit trusts delegate administrative (e.g., provider contracting) and/or financial functions (e.g., claims management) to contracted implementation support agencies (ISAs), such as third-party administrators (TPAs; which are private agencies registered under the government to provide support to health insurers) [25] and/or an insurance company (IC). These arrangements largely reflect a state’s operational capacities and prior PFHI implementation experiences.

#### State implementation models

Uttar Pradesh is implementing a model in which the SHA (the State Agency for Comprehensive Health and Integrated Services) contracts four TPAs to support PM-JAY roll-out (**TPA Model**). While the state had previously implemented an earlier PFHI (i.e., Rashtriya Swasthya Bima Yojana (RSBY) from 2008 to 2015), there were no active schemes to be integrated under PM-JAY. The estimated number of PM-JAY-eligible households is 124 million, the largest in our three states.

In Chhattisgarh, the SHA (the State Nodal Agency) contracts a private IC (IC Model), with a TPA internally assisting the SHA. PM-JAY roll-out subsumed the RSBY operating since 2009, which offered coverage for the poorest. Furthermore, the Mukhya Mantri Swasthya Bima Yojana (MSBY) has operated since 2012 and covers the remaining population, thereby providing universal insurance coverage in the state. Both schemes are overseen by the SHA, and use the same staff and resources and administrative and financial processes. The estimated number of eligible households under both schemes is 27.8 million.

In Tamil Nadu, the SHA (the Tamil Nadu Health Systems Project) contracts a publicly owned IC, which further sub-contracts three TPAs to process claims and provide administrative support (Hybrid Model). The Chief Minister’s Comprehensive Health Insurance Scheme (CMCHIS) was introduced in 2011 for households earning less than INR 72,000 annually. Approximately half of households deemed eligible by the CMCHIS income criteria did not meet PM-JAY eligibility criteria; the state continued to cover these households through additional state funding. The estimated number of households for both eligible groups is 14.7 million.

All three models established similarly functioning hierarchical structures to facilitate administration of PM-JAY. Internal information technology and program support teams assist SHAs in administration. SHAs are linked to administration officials responsible for scheme oversight within districts. A district implementation unit (DIU) is responsible for providing support to hospital functionaries for scheme processes. Depending on the model, the IC and TPA also have corresponding staff for district administration and implementation. Within hospitals, a hospital administrator, designated medical officer, and insurance navigators know as Ayushman Mitra (AM) are responsible for beneficiary assistance, assisted by data managers who perform information technology (IT)-related functions such as patient registration, pre-authorization requests, medical record-keeping, and claims processing. While the three implementation models utilize slightly different administrative structures, entities within these structures perform analogous functions aligned with national PM-JAY guidelines. Further state characteristics and model details are summarized in Table 1.

**Table 1:** Program design and implementation structure in the Pradhan Mantri Jan Arogya Yojana (PM-JAY)

PM-JAY Program Model	TPA Model	IC Model	Hybrid Model
Implementation state	Uttar Pradesh	Chhattisgarh	Tamil Nadu
Mode of implementation	Trust	Mixed mode	Mixed mode
Name of state health agency (SHA)	State Agency for Comprehensive and Integrated Services	State Nodal Agency	Tamil Nadu Health Systems Project
Implementation support agency: insurance company (IC)	None	1 IC	1 IC
Implementation support agency: third-party administrator (TPA)	4 TPAs	1 internal TPA (only to support high-value claims)	3 TPAs
Previous or pre-existing scheme (including year of termination, if applicable)	RSBY, terminated in 2015	RSBY, for below-poverty-line population, terminated immediately before PM-JAY launch; MSBY, continuing for all of the non-PM-JAY-eligible population	CMCHIS, continuing for some of the non-PM-JAY-eligible population
Eligible households for PM-JAY and currently implemented state schemes (September–December 2021)*	124 million	27.8 million	14.7 million
Geographic location	Northern India	Central India	Southern India

^*^Source: Information compiled by authors from https://nha.gov.in/PM-JAY Accessed on 24.11.2022

### Conceptual approach

To explore whether and how implementation experiences are shaped by the different PM-JAY arrangements and contextual features for each model, we adopt a conceptual framework developed and validated by the National Implementation Research Network to identify key drivers of program implementation [26]. This framework characterizes essential design components and activities based on their contributions to “drive” the implementation process: competency drivers contribute to ensuring that involved actors can acquire all relevant skills and qualifications needed for program implementation, organizational drivers contribute to the overall alignment and coordination of implementation activities, and leadership drivers contribute to overarching implementation supervision and management. Sub-aspects pertaining to each of these implementation drivers are described in more detail in Table 2. We selected this framework because it seeks to understand factors influencing implementation outcomes retrospectively, and could be simultaneously applied to different implementation strategies in each state [27]. We additionally explored contextual elements influencing these drivers and implementation within each state [28].

**Table 2:** Competency, organizational, and leadership drivers affecting program implementation

Implementation drivers		Definitions
Competency drivers	Coaching (on-job)	Key activities pertaining to competency of different program implementers
	Training (education)	
	Selection (of human resources)	
	Facility selection	Key material/equipment/qualifications that determined how facilities were selected to implement the program
	Performance assessment	Key features used to monitor and address performance of relevant implementers
Organizational drivers	Systems-level intervention	Key features that define how the program and its implementation is integrated within and across relevant systems
	Facilitative administration	Key administrative features that support program implementation
	Decision support data system	Key technical features that allow decision-making during the implementation process to be data-driven
Leadership drivers	Technical	Key personnel and activities that ensure the technical oversight of the implementation process
	Adaptive	Key personnel and activities that ensure implementation processes to adapt to identified challenges and constraints

Source: Based on Bertram et al. [26]

### Study design and data collection

Our qualitative study adopts an exploratory approach for which we conducted 92 in-depth interviews (IDI) with different central-level and state stakeholders between February–April 2019 (Table 3). At central level, we conducted 15 interviews (reaching theoretical saturation) with purposely selected individuals who played a key role in the design and early implementation of PM-JAY, including: government program designers and implementers, NHA officials, multi- and bi-lateral development partners, an insurance representative, and civil society/academics. All central respondents were approached by the study team through e-mail communication. One central-level interview was not completed due to other commitments of the respondent (and hence, not included in the final interview count).

**Table 3:** Interview respondents

Respondent category	Level (*n*)			
	Central	State		
		Chhattisgarh	Tamil Nadu	Uttar Pradesh
Government officials	4	3	1	2
Development partners	8	1	NA	2
Academic/civil society	2	–	–	–
Insurance representative	1	1	1	NA
Consultancy firms/third-party administrators	NA	1	3	3
District functionaries	NA	4	4	5
Hospital administrators	NA	17	12	17
Total	15	27	21	29

NA, not applicable

Within states, two districts were identified (anonymized for confidentiality) whose implementation progress was considered better and average among all districts, according to the respective SHAs. The study team first approached the SHA to obtain requisite permissions; the SHA then facilitated contact with all state respondents, including within the SHA, district functionaries, and hospitals. Within each district, we sought to conduct at least six interviews with administrators of public and private empanelled hospitals. The final number of district functionaries and hospitals reflected the availability of staff and facilities within districts, and all approached respondents consented to the interviews. In total, we conducted 18 interviews with members of SHAs and respective ISAs and 13 interviews with implementation officials at the district level in the three states. Finally, we conducted 46 interviews with hospital administrators of public and private hospitals empanelled in PM-JAY in these districts. All respondents except development partners and academic/civil society (Table 3) were directly involved in the implementation of PM-JAY.

For each respondent category, we used a different, contextually adapted semi-structured interview guide to explore aspects related to PM-JAY design and implementation within states and districts, focusing on how national PM-JAY guidelines had been adapted to the local context and the structure of the selected model. Content covered in each guide was adjusted to the local context and implementation model. In-person interviews were conducted during working hours in the respective official workplaces of all respondents and lasted for approximately 1–1.5 h each. SS and MDA conducted all central-level interviews, and SS conducted all interviews at state level, either in English or Hindi. Interviews within districts were conducted by trained staff in English or Hindi, and supported by bi-weekly debrief sessions with SS. Written informed consent was obtained from all respondents. Interviews were audio recorded, transcribed verbatim, and translated into English. Three respondents refused to be recorded during interview, but agreed to note-taking.

### Analytical approach

We used a deductive approach to content analysis by coding and mapping interview content for every aspect under each implementation driver in Table 2. This coding matrix was expanded to include additional codes indicating any relationships to earlier PFHI implementation, contextual elements, and specific qualities “facilitating” or “hampering” the overall implementation process, within a constructivist research paradigm. Two researchers coded and visualized data using NVivo software [29] in an iterative process, accompanied by discussions to ensure coding alignment. Coded information was triangulated and interpreted separately for each implementation model, to identify linkages between organizational features, drivers, and contextual elements affecting implementation.

The study adheres to the Standards for Reporting Qualitative Research guideline [30].

## Results

We present how each driver (Table 2) affected implementation of PM-JAY, first describing similarities across models, followed by model-specific findings. Quotes from respondents illustrating key findings are presented in Table 4.

**Table 4:** Selected quotes from respondents

**Competency drivers**
Selection of human resources: “So, we had to employ Ayushman Mitras according to the hospitals, we had to deploy 2 Ayushman Mitras in hospitals with more than 50 beds. So, we conducted the interviews of all the Ayushman Mitras before 16th September and we didn’t take previous employees as all of them were corrupted. That is why we took fresh employees whom we would be able to understand better because the process of PM-JAY and RSBY is totally different. So, it is very difficult for the previous employees to forget about the previous process and implementing the new process, but it was easier to implement the same thing with the help of newer employees. That is why we hired new employees who could work and who had the will to work.”—State Health Agency respondent, male, age 44 years, 20 years’ work experience, Chhattisgarh
Selection of human resources: “Under the CMCHIS we have appointed some special position in some department which leads to overall improvement but they have paid very minimal money. I think they are paid very less money.”—State Health Agency respondent, female, age 40 years, 13 years’ work experience, Tamil Nadu
On-job coaching: “No, training was given, in fact it was just a formality…There are only two people in all government hospitals in XXX, one is XXX and one is District level and no other block is involved. In winters everyone sits around the fire. In the training they had just shown a presentation.”—Hospital respondent, male, age 40 years, 12 years’ work experience, Uttar Pradesh
**Organizational drivers**
On attitude of support team: “Working team has not been here to support us. They have been here to find out our mistakes and how to reject cases.”—Hospital respondent, male, age 53 years, 23 years’ work experience, Chhattisgarh
On medical paternalism and relationships between different doctor cadres: “We had a meeting in IMA [Indian Medical Association] and there we complained about the support team. They can’t raise their finger on a surgeon or radiologist or gynecologist when they themselves are BAMS or BHMS because they have no idea about surgeries. At-least the doctor from X [insurance company] should be MBBS and the District Manager should have some technical knowledge and they should cooperate with us because we are treating patients.”—Hospital respondent, male, age 47 years, 16 years’ work experience, Chhattisgarh
On relationship between hospitals and TPAs: “…that is the main issue because there is the problem. Definitely there is a problem. The third party wants to be more autonomic. The third party wants to be not synchronizing with the medical team and maybe they think that they may not know that the medical persons and they may not be knowing on this, so they can decide their own. So those attitudes should be completely [gone], it is very important.”—Hospital respondent, male, age 58 years, 28 years’ work experience, Tamil Nadu
Inadequacy of reimbursement rates: “I do not see much benefit, and actually financially, we have the support from various other sources because the amount provided by the government is not enough, especially in complicated cases it is very, very difficult to manage.”—Hospital respondent, male, age 60 years, 30 years’ work experience, Tamil Nadu
On organizational culture: “This organization is a public organization. So, the way of working here is like public organizations in UP.”—State Health Agency respondent, female, 5 years’ work experience, Uttar Pradesh
**Leadership drivers**
On state wanting greater leadership: “Many things are handled by NHA which need to be given to the state level authority that can be easily handled. For every small thing we need to generate a ticket, we keep on requesting via email to NHA or X [information technology company], for doing any small changes also.”—State Health Agency respondent, male, 1.5 years’ work experience, Chhattisgarh
On state leadership based on prior expertise and legal authority: “And the certain things were, see legally speaking we emphasis state subject here… So, naturally we will have a better hold over the scheme implementation and second thing is, our is a little bigger scheme and older scheme. So naturally we will also try to maintain whatever the technical and political gain, which comes along with the scheme.”—State Health Agency respondent, male, 30+ years’ work experience, Tamil Nadu
On coordinated leadership: “Mr. XX has been involved since the beginning and he has been one IAS [Indian Administrative Service] officer who has every update and every health scheme and PM-JAY being the flagship program, he has facts and figures on his fingertips. I think he calls and talks to Mrs. XX at least once a day, there is a conference call with all the CMs [Chief Medical Officers] and DMs [District Magistrates] once a week, there is a video conferencing and meeting with the chief secretary once a week, so there is a lot of updates, there is a meeting with CM every month, so that is a lot of monitoring, that is a lot of handholding, lots of cautions from seniors which is a good thing. But if you come to the mid-level which is comprised of people like directorate, then have been equally involved.”—State Health Agency respondent, female, 15 years’ work experience, Uttar Pradesh

### Competency drivers

All three models adapted PM-JAY national guidelines for the recruitment and competency development of all staff required for hospital empanelment and subsequent implementation. The models exhibited some commonalities. All states were required to empanel public hospitals irrespective of whether they fully met empanelment criteria, to ensure similar geographical access to basic tertiary services. Hospital respondents reported increasing manpower to meet procedural and service demands under PM-JAY. Public hospitals hired contractual staff for patient management, data management, and nursing; private hospitals hired similar staff, but reported that the low reimbursement rates for specialized services were not amenable to contracting specialist doctors to perform these services. PM-JAY-specific trainings were greatly needed, as common pre-service qualifications of hospital staff did not support non-clinical aspects of PM-JAY implementation. Oftentimes there was a disconnect between the relevance of promoted on-job skill development activities and how these were perceived by staff. Most hospital functionaries undergoing skill-development activities reported great scope for improvement, especially for key PM-JAY-specific processes such as beneficiary identification and claims processing.

All three models reported structured processes for performance assessment, including audits and feedback meetings, which were overseen by SHAs and assisted by TPAs/IC. SHA officials reported that it was too early after PM-JAY adoption to assess performance, as the scheme was not fully operationalized. However, all three SHAs viewed PM-JAY contracts as a favorable means to regulate and strengthen the capacities of empanelled hospitals (which were initially quite low, especially in remote districts) while increasing access to critical services. Very few hospitals used any PM-JAY-related performance assessment data as feedback into hospital functioning, except for profit–loss monitoring. Hospitals were dissatisfied with administrative support and claims processing and reimbursement, and sharply critiqued the benefit package and package rates. They overwhelmingly perceived that PM-JAY was positively affecting beneficiary awareness and utilization, but beneficiary identification data issues lead to many well-off households availing benefits, creating an “entitlement mindset,” while excluding deserving households.

#### TPA model

In Uttar Pradesh, the state agency previously implementing RSBY was re-engaged for PM-JAY. However, most SHA personnel had to be hired afresh, with the exception of some higher-level staff. Hiring processes were often impeded by challenging bureaucratic regulations. Technical and managerial support to SHA personnel was provided through a project management unit comprising contractual staff, with some staff recruited by consultancy firms contracted by the SHA for scheme management and beneficiary outreach activities, and some staff funded and recruited independently by development partners. Despite these added resources, SHA employees reported being understaffed and fulfilled multiple job roles.

While the SHA adhered to national guidelines for empanelling providers, these were loosely enforced as many districts did not have adequately equipped hospitals. Within districts, the SHA established a decentralized process to improve efficiency and reduce political interference with staff selection. Within each district a medical doctor/district coordinator, information systems/data manager, and grievance manager (together functioning as the DIU) were selected by the chief medical officer and other district officials, and further supported by a contracted agency. However, entrenched medical hierarchies affected hiring of medical doctors for the DIU. Medical graduates were unwilling to work in districts for the proposed remuneration, and the SHA fulfilled this shortage by appointing non-medical staff, or doctors with Indian Systems of Medicine (ISM) qualifications (i.e., doctors trained in traditional Indian medicine versus biomedicine) to DIUs. ISM doctors accepted these roles, as they provided augmented remuneration and job opportunities. Doctors from empanelled hospitals did not regard these staff well and perceived them to be underqualified.

District teams reported shortages of data managers. Public hospitals reported constraints in beneficiary processes as AMs were not placed there. Hiring AMs was also challenging for private hospitals, as prescribed remuneration amounts were too low for the mandated professional qualifications and working hours. Further, many hospitals were initially hesitant to empanel due to prior unfavorable experiences with RSBY and prohibitive documentation requirements and low reimbursement rates in PM-JAY.

SHA officials favorably assessed their performance on claim processing, verification of beneficiaries, and timely empanelment and capacity development of hospitals. The relative performance of the four TPAs and districts assigned to them was regularly assessed. District officials felt that beneficiary identification was the biggest challenge, and once streamlined, would greatly improve performance. The majority of hospitals conducted minimal performance assessment activities and had limited programmatic interactions and feedback.

#### IC model

In Chhattisgarh, the government agency overseeing MSBY and RSBY was additionally entrusted with SHA functions for PM-JAY. The SHA included core personnel, an IT team, and contractual staff assisting in claims processing, which was further supported by the IC. Claims processing for high-value claims was performed by the internal TPA and a few staff funded and placed by a development partner. While hospital empanelment under RSBY and MSBY was initially delegated to the IC, the SHA now performed this function for better procedural oversight. Additionally, minimum bed strength and human resource empanelment criteria had to be relaxed in certain districts, as there were inadequate facilities meeting these criteria.

Government hospitals reported acute staff shortages both for medical officers, specialists, and PM-JAY dedicated staff. Many government hospitals tried to compensate shortages by hiring contractual staff (especially surgeons and administrators). Contractual staff faced issues regarding established staff hierarchies and working relationships, with contractual staff not regarded as equals by regular staff, or given tasks refused by permanent staff.

Under both RSBY and national PM-JAY guidelines, AMs were to be hired directly by hospitals. AMs were not recruited for government hospitals. For private hospitals, the SHA mandated that AMs be hired and supervised by the IC to reduce incentives for AMs to collude with hospitals. AMs would thus adhere to standard operating procedures under IC supervision, rather than being subject to variable standards under each hospital. Further, AMs employed under previous schemes were not retained to limit the “institutional memory” of previous schemes to negatively impact PM-JAY implementation. Instead, new AM personnel were hired; this was challenging because the low remuneration did not attract sufficiently qualified staff. Once recruited, AMs were reportedly not well received by hospitals.

SHA and district officials were appreciative of IT systems, high volume and timeliness of claim authorization, and increasing capacities of hospitals to implement PM-JAY over time. However, many hospital respondents could not differentiate between activities for PM-JAY and MSBY. Many hospitals conducted minimal performance assessment activities and had limited programmatic interactions and feedback.

#### Hybrid model

In Tamil Nadu, staff working for CMCHIS were further entrusted with PM-JAY. The SHA functioned as a project team within the larger Department of Health, supported by a government-owned IC, three TPAs, and an IT team. Staff recruitment and training as well as facility empanelment followed stringent processes established under CMCHIS. The state introduced mandatory accreditation criteria for private hospitals (National Accreditation Board for Hospitals & Healthcare Providers) and public hospitals (National Quality Assurance Standards). This required hospitals to obtain the relevant certification within a year of empanelling, failing which they would be de-empanelled. Tamil Nadu was currently ratifying the Clinical Establishments Act, under which all hospitals would have to compulsorily register; this would then additionally be mandatory for empanelment.

CMCHIS’ efficient administrative structures within districts and hospitals additionally executed PM-JAY responsibilities. AMs were hired and placed within hospitals by the IC, to maintain their independent functioning. Within hospitals, remuneration rates for designated staff under CMCHIS were too low, and hospitals were hopeful that PM-JAY would remedy this.

SHA officials reported no change in the status quo from before PM-JAY implementation and remarked that everything was functioning as before. Hospitals could not distinguish between performance assessment activities for PM-JAY and CMCHIS.

### Organizational drivers

As previously described, all three models established similarly functioning hierarchical structures and a cascade of standardized processes aiding monitoring and implementation, aligned with national PM-JAY guidelines. These largely reflected the state’s institutional memory of earlier or co-existing PFHI. SHA respondents appreciated the NHA’s role as the apex facilitative administrator, with open exchanges and “human connections” among individuals.

SHA, district, and hospital respondents in all models faced uniform challenges with organizational and administrative processes. Data challenges included obsolete or incomplete data used for determining beneficiary eligibility; mismatches in hospital empanelment data at NHA and SHA levels; inability to link data between hospital empanelment and claims processing data systems, which operated on different technology platforms; missing information for specific benefit packages and their procedural requirements; and technicalities such as software issues, faulty IT servers, and poor internet connectivity.

Hospital respondents reported that inconsistencies in PM-JAY data systems and architecture frequently resulted in delayed timelines, resulting in patient inconveniences, inefficiencies, and claim rejection. Guidelines necessitated that beneficiary verification procedures and pre-authorization requests to the SHA for patient admission were to be performed by AMs round-the-clock; however, AMs were only posted for 8-h shifts and not available 24/7. Similarly, medical doctors required for sanctioning pre-authorization requests or for discharging patients were not available 24/7.

Once electronic pre-authorization requests were placed by AMs, support teams within the SHA, TPA, or IC engaged with AMs or data managers within hospitals to ensure timely compilation of patient medical records and documentation for claims processing. However, hospitals were dissatisfied with services provided by support teams, citing a lack of frank communication, information asymmetries, and an overtly critical attitude. Entrenched medical hierarchies hampered communication in all models, affecting interactions between AMs, medical officers, district officers, and higher authorities. Medical doctors in hospitals repeatedly questioned the ability of SHA, TPA, or IC staff without similar clinical qualifications to scrutinize their treatment plans or authorize claims. ISM doctors assisting claims processing in TPAs reported being regularly questioned and challenged in their roles by hospital medical doctors.

Most respondents questioned the adequacy of the benefit package and its reimbursement (package) rates, even after all models adapted the nationally recommended benefit package to state requirements. This was a lesser issue in government hospitals, which additionally received line budget funding. Hospital respondents described gaps in the benefit package for chronic diseases, co-morbidities, and surgical treatments. Benefit packages did not cover some commonly prescribed drugs, resulting in out-of-pocket patient expenditures. Reimbursement rates for most packages were too low and below industry standards; respondents highlighted specific packages such as cataract, which did not cover intraocular lens costs, and for orthopedics, which did not cover prostheses costs.

Hospitals reported multiple inefficiencies with claims processing, including unclear and cumbersome documentation requirements, inadequate and delayed support from TPAs and support agencies, delayed and incomplete payments, and unjustified claim rejections. All of these endangered the financial viability of hospitals and thus affected all other implementation drivers, and impeded the provision of quality services. Respondents from hospitals, TPAs, and SHAs remarked that the claims reimbursement procedure followed a learning curve, with all stakeholders increasing efficiency and timeliness over time.

#### TPA model

The TPA model followed national PM-JAY organizational and process guidelines with minimal adaptations. This was attributed to the state’s earlier, unfavorable experiences with RSBY, which created a cautious perception for PHFI, including among hospitals and beneficiaries. Beneficiaries’ fear of being forced to pay for services in private hospitals and a deep distrust of services in public hospitals further motivated the SHA’s cautious approach. The SHA entirely re-scrutinized and subsequently authorized claims processed by the TPAs. Respondents stated that this stemmed from the state’s poor experiences with claims processing under RSBY, and ongoing legal disputes between the government and hospitals regarding RSBY claim settlement. However, the TPAs felt disempowered by this. Double scrutiny of claims was also motivated by poor regulation and oversight over private providers. State and district respondents reported that the bureaucratic governance style hampered implementation. Unequal salary structures between the SHA and district teams created conflicting inter-personal hierarchies, as some district staff were paid substantially more than SHA staff with similar or higher designations. Lack of computerized record-keeping in public hospitals and poor internet connectivity in many districts were further challenges.

#### IC model

The IC model organization reflected state capacities in implementing RSBY and MSBY; many national PM-JAY processes were adapted to those in these schemes. IT and data-reporting systems, benefit packages, and claim processing were all adapted to state requirements. Under MSBY, claims processing was divided between the IC and the SHA, which continued in PM-JAY: claims below INR 50,000 were processed by the IC (approximately 90% of all claims), while those of greater value were processed by the SHA assisted by the TPA. Despite the larger volume of claims processed by the IC, the SHA exercised considerable decision control. Some respondents brought up challenges of working in a government system, with a reluctance from state leadership to accept programmatic problems, and poor regulatory oversight of private providers. Further, the incumbent state government had an opposing political ideology to the national government, and respondents speculated about the future PM-JAY in the state. SHA representatives voiced a need for greater autonomy from the NHA, especially for routine administrative decisions. Lack of computerized record-keeping, insufficient staff in public hospitals, and poor internet connectivity in most districts were further challenges. Since almost the entire population in Chhattisgarh was eligible for MSBY, many beneficiaries were reportedly confused about PM-JAY entitlements and targeting.

#### Hybrid model

The Hybrid model incorporated in entirety the organizational structure, IT and data platforms, beneficiary identification data, and standardized operating procedures of CMCHIS. The model greatly modified the national PM-JAY benefit package for organizational efficiency: many secondary and all preventive procedures were reserved for public hospitals, as they could be accessed cost-free within the public system, and state officials did not want a dual system of basic service provisioning. These reserved packages constituted nearly 50% of the volume of utilized packages. Benefit packages with reimbursement amounts less than INR 150,000 were wholly processed by the TPAs and reimbursed by the IC; those above this amount were approved and reimbursed by the SHA. Thus, both TPAs and the IC exercised considerable autonomy. The model strictly monitored performance indicators and employed a benchmarking system to grade hospitals using selected criteria; claims reimbursement rates were tiered according to these assessments as financial incentives for quality service provision. A district vigilance officer in each district monitored hospital activities and resolved beneficiary grievances. Since PM-JAY eligibility criteria covered only half of the CMCHIS-eligible population, administrators viewed PM-JAY to be a “subset” of it. Tamil Nadu’s political leadership was different than the national government, and the SHA emphasized the need for continuing operational autonomy.

### Leadership drivers

Respondents across models appreciated the unique, specialized technical leadership capacities of the NHA, affirming that these were unusual within government agencies. Respondents further appreciated adaptive leadership roles played by high-ranking SHA officials, and collaborative leadership among state and district health administration staff. Political leadership within the respective states was reported to affect leadership styles within the three models. States with the same political leadership as the national government (TPA model) adopted the national guidelines almost in entirety, while states with different political leaderships (IC and Hybrid models) were more entrepreneurial with PM-JAY design flexibilities. Leadership styles within models were also reflective of their prior experiences with PFHI and bureaucratic systems; respondents in the TPA model reported a cautious leadership approach due to unsatisfactory experiences under RSBY and high politicization of the scheme, whereas leadership in both IC and Hybrid models was more independent, reflective of their long-standing PFHI implementation expertise. However, in all three models, government bureaucracy and regulatory inertia reportedly limited leadership potential.

## Discussion

Our analysis illustrates how competency, leadership, and organizational drivers shaped the implementation of PM-JAY in three contextually different Indian states. While design flexibilities resulted in a “different” PM-JAY model being implemented in each state, this was not only possible but also necessary under Indian federalism, and it leveraged existing competencies in a decentralized health implementation space, e.g., through state PFHI implementing agencies. Model adaptations were also needed to tailor implementation to local capacities, such as by adjusting recruitment or empanelment criteria. These complexities pose challenges in assessing comparative model performance. Since our study was not designed to assess outcomes (which would necessitate quantitative approaches), we infer where drivers enabled or hindered effective functioning based on respondents assessments [27, 31, 32]. Further, since data collection occurred approximately 6 months after the adoption of PM-JAY, our results represent early implementation realities.

Each model used contextually relevant adaptations for facility selection to facilitate implementation. However, hospitals everywhere reported that meeting selection criteria to empanel in PM-JAY was often not worthwhile or profitable. Staff selection criteria varied across models based on state capacities, to enable hiring of relevant, available staff. Despite these adaptations, conflicts with other aspects of staff selection or reimbursement guidelines created challenges with hiring, contracting, or retaining staff, especially medical specialists who were the mainstay of clinical staff, and AMs, who were critical for patient navigation. These problems were acute in public hospitals, which had higher patient loads and inadequate or no designated staff for PM-JAY. This warrants special attention since public facilities constituted more than half of all PM-JAY empanelled facilities in 2020, especially in Tamil Nadu and Uttar Pradesh [33]. Moreover, in-service coaching to equip staff with necessary skills were hastily done and inadequate. Similarly, pre-service training of staff supported clinical competencies but not for non-clinical program implementation aspects. These findings highlight the need to better align the joint needs and competencies of the scheme, and of staff and hospitals implementing the scheme.

Respondents reported mixed experiences with structured (but functional) facilitative administration processes. While all models exhibited gradual improvements over time, immediate barriers included obsolete data for PM-JAY beneficiary identification, inconsistencies in data systems, and IT issues (especially in public hospitals). District implementers in the TPA and IC models reported unsatisfactory engagement with higher-level program functionaries. Hospitals in all models reported inadequate facilitative support from designated support teams due to poor capabilities and communication skills. Procedural inconsistencies like shorter contracted working hours resulted in the lack of availability of AMs or doctors to provide facilitative support 24/7 in hospitals, which contradicted scheme guidelines. All respondents recognized that benefit packages and reimbursement rates needed urgent revision. Nearly every aspect of claims processing was beset with problems and impeded the provision of quality services. These issues have also been reported from recent Indian studies on PM-JAY [9–11, 33]. Performance assessment activities pertained to the performance of the overall program (rather than of individual staff or hospitals) and reflected differences in program models. Barriers for performance assessment activities included variable data reporting structures, with a near absolute lack of performance assessment activities for PM-JAY within hospitals in Chhattisgarh and Uttar Pradesh. This is emblematic of the overall lack of information culture and data usage in Indian PFHI [34] and weak regulatory and contextual structures enforcing quality improvement and accountability [35]. PM-JAY has tried to remedy this with national-level data reporting, but the lack of feedback loops to hospitals indicates that more can be done to institutionalize data use for decision-making, transparency, and accountability [36].

Some model-specific adaptations greatly aided implementation efficiency. In IC and Hybrid models, splitting claims processing between the IC and SHA improved efficiency and timeliness while fostering a healthy inclusiveness among, and empowerment of, collaborating agency staff. However, the efficiency of TPAs for claims processing has been questioned in other Indian PFHI [37]. Decentralizing staff selection and recruiting alternate cadres of health workers in the TPA model enabled PM-JAY to function in areas of acute human resource shortages. The Hybrid model utilized the most stringent empanelment, accreditation, monitoring, and benchmarking criteria to regulate hospital and program performance, first under CMCHIS and later extended to PM-JAY [38]. These strategies have seen success in better-regulated health systems such as Europe [39] and transitioning economies such as Chile, Mexico, and Vietnam [40]. Further, reserving secondary care benefit packages for public hospitals ensured an efficient utilization of public funds; subsidization of services in private hospitals, which are available in the public health system, has been a major critique of Indian PHFI [35, 41, 42]. A more systematic study of these adaptations and their contributions to implementation outcomes could help promote important policy-transfer to other locations and scalability of these adaptations [38, 43, 44], and advocate for improved regulation of health providers.

Model-specific adaptations also resulted in unintended consequences. In all models, medical paternalism impaired interactions between medical doctors, ISM doctors, and non-clinical staff. This impeded recruitment and retention policies, leading to governance challenges and poor accountability [45]. Remuneration practices in the TPA model, which compensated district staff higher than SHA staff, created structural barriers in staff interactions while simultaneously not attracting sufficiently qualified staff. Contractual staff hired in public hospitals were reportedly regarded as “second-class” employees by peers. AMs in the IC model perceived that they were “outsiders” in the hospitals in which they worked, as a consequence of being hired by the IC. These officially sanctioned guidelines challenging existing normative, social, and power hierarchies may have led to discretionary practices at the “local” or facility level by agency-constrained staff seeking to navigate them, resulting in less than optimal implementation [46, 47]. While these are systemic issues, greater attention is needed toward staff recruitment, implicit power asymmetries, and organizational culture [45, 46, 48].

Multi-dimensional contextual elements affected implementation drivers in all models, underlining the importance of context [5, 49]. The most profound of these was states’ previous experiences with PFHI, which affected all involved stakeholders, including SHAs, district functionaries, hospitals, and beneficiaries. Model implementation features further mirrored the institutional memory and underlying capacities of previous schemes in all aspects. Innovative adaptations in the Hybrid model were enabled by the availability of sufficient hospitals, human resources, and a robust governance and institutional structure [33, 50], and may not have worked elsewhere with service delivery gaps [51] and different contexts [39]. The type of political leadership at the state and national levels and complex government bureaucracy created an organizational culture that hindered adaptive leadership approaches. For example, the TPA model followed national PM-JAY guidelines to the greatest extent among the three models, stemming from a general lack of PFHI capacity and allegiance with the national leadership. These contextual elements also affected beneficiary behavior and acceptance of PM-JAY, with a cautious, skeptical approach in the TPA model to robust enthusiasm in the others.

### Limitations and suggestions for future inquiry

Our study also has some limitations. The first set of limitations pertains to qualitative studies. Purposive sampling may have led to the omission of key stakeholders who were not interviewed; we could not interview AMs who were directly responsible for implementation, as it was not possible to do so without causing disruptions to their heavy workload. We cannot rule out recall or participant biases among respondents, which we have addressed by triangulation of responses across the respondent groups [52]. The second set of limitations pertain to the choice of analytical framework, which was pragmatically motivated to address the three disparate program models. As the specific details of program implementation models in the three states were adaptable and program activities not explicitly defined a priori, we could not assess the fidelity of each program implementation model. We also faced the challenge of greater prioritization of some drivers by respondents—e.g., many respondents placed a great emphasis on organizational drivers, power dynamics, or organizational culture, and it would be useful to examine these in greater detail on their own. Further, as the study was conducted in the initial implementation stage, we could not link the functioning of the implementation drivers to program outcomes. This is an area for future research, as outcomes information will become available as the program matures and achieves full implementation. Additionally, comparisons between the functioning of the drivers between the full and initial implementation stages will facilitate understanding the mechanisms of change and areas for further improvement. Lastly, program beneficiaries were not the focus of our study, and understanding their perceptions of and experiences with the program, and how the program addresses these, will be crucial to implement PM-JAY in a manner that achieves improved population outcomes.

## Conclusions

PM-JAY guideline flexibilities enabled implementation in very different states through state-adapted implementation models. These models utilized contextually relevant adaptations for staff and facility competencies and organizational and facilitative administration, which had considerable scope for improvement in terms of recruitment, competency development, programmatic implementation support, and rationalizing the joint needs of the program and implementers. Adaptations also created structural barriers in staff interactions and challenged implicit power asymmetries and organizational culture, indicating a need for aligning staff hierarchies and incentive structures. At the same time, specific adaptations like decentralizing staff selection and task shifting (all models), sharing of claims processing between the IC and SHA (IC and Hybrid model); use of stringent empanelment, accreditation, monitoring, and benchmarking criteria for performance assessment; and reserving secondary care benefit packages for public hospitals (both in the Hybrid model) contributed to successful implementation. Contextual elements such as institutional memory of PFHI and underlying state capacities influenced all aspects of implementation, including leadership styles and autonomy. These variations make comparisons across models difficult, yet highlight constraints and opportunities for cross-learning and optimizing PFHI implementation to achieve UHC in decentralized contexts.

## Data Availability

The data that support the findings of this study are available from Deutsche Gesellschaft für Internationale Zusammenarbeit GmbH, but restrictions apply to the availability of these data, which were used under license for the current study, and so are not publicly available. Data are however available from the authors upon reasonable request and with permission of Deutsche Gesellschaft für Internationale Zusammenarbeit GmbH.

## Abbreviations

AM: Ayushman Mitra
CMCHIS: Chief Minister’s Comprehensive Health Insurance Scheme
DIU: District implementation unit
IC: Insurance company
IDI: In-depth interview
ISA: Implementation support agency
ISM: Indian System of Medicine
IT: Information technology
MSBY: Mukhya Mantri Swasthya Bima Yojana
NHA: National Health Authority
PFHI: Publicly funded health insurance
PM-JAY: Pradhan Mantri Jan Arogya Yojana
RSBY: Rashtriya Swasthya Bima Yojana
SHA: State Health Authority
TPA: Third-party administrator
UHC: Universal health coverage
